# An umbrella review for preoperative rehabilitation in primary total knee arthroplasty: quality assessment and summary of evidence

**DOI:** 10.1186/s12891-025-08541-y

**Published:** 2025-07-04

**Authors:** Yan Zhao, Chong Tian, Songxin Tian, Wei Han, Hui Shi, Mingyu Cao, Jie Liu

**Affiliations:** 1 Department of Rehabilitation Medicine, the Sixth Affiliated Hospital of Xinjiang Medical University, Urumqi, 830000 Xinjiang China; 2 Department of Rehabilitation Medicine, the First Affiliated Hospital of Xinjiang Medical University, Urumqi, 830054 Xinjiang China; 3 Department of Rehabilitation Medicine, People ’s Hospital of Xinjiang Uygur Autonomous Region, Urumqi, 830092 Xinjiang China; 4 Department of Bone and Joint Surgery, the Sixth Affiliated Hospital of Xinjiang Medical University, Urumqi, 830000 Xinjiang China

**Keywords:** Preoperative rehabilitation, Total knee arthroplasty, Evidence summary, Umbrella review

## Abstract

To evaluate the effects of preoperative rehabilitation in primary total knee arthroplasty patients and create an umbrella review. Web of science, PubMed, Epistemonikos, and Cochrane Library were searched from inception to 30 September 2023. This study combined the terms of total knee arthroplasty, and preoperative rehabilitation to retrieval relative systematic reviews (SR) and meta-analyses (MA). The 2 reviewers independently completed article screening, data extraction, and measure of methodological rigor quality. A Measurement Tool to Assess Systematic Reviews (AMSTAR-2) was used to measure the methodological quality of the included SRs. A total of 14 eligible SR and MA were eventually included in the qualitative analyses. The AMSTAR-2 indicated that the quality evaluation grade of 9 articles was ‘Critically low’ and the rest were ‘Low’. The findings indicate that exercise and physiotherapy, which are the primary preoperative rehabilitation measures, exhibit a negligible impact on postoperative pain and functional enhancement. Two studies of Meta-analyses combined with RCTs provided strong evidence that preoperative rehabilitation significantly reduces hospital length of stay by pooling data from multiple RCTs, and the results were statistically significant. Two other systematic reviews that pooled the results of existing studies also supported that preoperative rehabilitation reduces hospital length of stay. Nevertheless, the preponderance of studies proved inadequate in substantiating enhanced functional prognoses with preoperative rehabilitation. There was considerable heterogeneity in the selection of interventions and outcome metrics across the studies. Future research needs to address these issues through appropriate doses and fully described interventions.

**Supplementary Information:**

The online version contains supplementary material available at 10.1186/s12891-025-08541-y.

## Introduction

Globally, the prevalence of knee osteoarthritis (KOA) in 2019 was about 364.6 million, and the disease burden of KOA increased greatly in most countries and regions recently [1]. Total knee arthroplasty (TKA) is one of the main therapeutic approaches for end-stage KOA [2]. KOA patients are often accompanied by pain, stiffness and decreased limb function. In recent years, the ‘rehabilitation’ program has attracted the attention of clinical staff because of its positive effect on improving patients’ pain, physical function and mental health [3]. Rehabilitation is patient-centered. Through psychological intervention, nutritional guidance and exercise intervention, it aims to optimize the preoperative physiological reserve, improve the functional level of patients, and accelerate the postoperative rehabilitation process of patients [4]. With the concept of enhanced recovery after surgery (ERAS) and the successful development of day surgery in orthopedics, the average length of stay of TKA patients is shortened, which makes the time of rehabilitation guidance move forward as a trend [5, 6].

However, according to existing research reports, the possible waiting time for operation not only imposes great burden on patients [7, 8], but also leads to pain, functional limitations, and deterioration of quality of life (which in turn affects postoperative outcomes) [9, 10]. The optimal dose of preoperative intervention can reduce preoperative physiological disorders and physical function deterioration, and may accelerate postoperative recovery [11]. At present, there is an expert consensus on preoperative management of knee joints, but no clear recommendations have been made on the content of rehabilitation, and there are still differences in the methods and outcomes of exercise intervention.

In this study, the best clinical evidence related to TKA rehabilitation was integrated and evaluated through a systematic literature search using an umbrella review approach. The main objective of the study was to provide a scientific basis for clinical staff to help them develop individualized rehabilitation interventions according to the specific conditions of different patients, in order to enhance postoperative recovery and promote functional recovery and quality of life improvement.

## Methods

### Guidelines

The literature search and the study reports were based on the Preferred Reporting Items for Systematic Reviews and Meta-Analyses (PRISMA) guidelines [12]. This study has been registered with PROSPERO under registration number CRD420250654961, details of which can be accessed: https://www.crd.york.ac.uk/PROSPERO/.

### Database and search strategy

A comprehensive literature search was conducted in the following databases: PubMed, Web of Science, Epistemonikos, and the Cochrane Library of Systematic Reviews and Meta-Analyses, covering the period from the inception of each database to 30 September 2023. The search strategy combined subject terms and free text terms focused on the following main themes: “total knee replacement,” “preoperative rehabilitation,” and “systematic reviews/meta-analyses.” A detailed account of the search strategy can be found in Appendix 1 (Supplementary Material Table S1).

In addition to database searches, we manually screened relevant literature by reviewing the reference lists of the included studies to identify additional sources that may have been overlooked by the search strategy.

### Study screening

The inclusion standards are as follows: (1) Population: the patients with primary total knee arthroplasty; (2) Intervention group: reported specific preoperative rehabilitation, such as exercise, education, physical modality therapy, and so on; (3) Comparison group: not specified; (4) Outcome measures: postoperative pain, physical outcomes, Quality of life, incidence of postoperative complications, length of hospital stay, etc.; (5) Study design: systematic reviews or/and meta-analyses; (6) Other: the search is limited to “human” and “English” studies reported in peer-reviewed journals. When multiple papers have reported the same outcomes, the more recent would be selected.

The exclusion standards are as follows: (1) Repetitive, unable to obtain the original text or incomplete information; (2) Literature as a guide to interpretation, meetings or proposals.

All titles and abstracts identified by the search strategy were reviewed by two evaluators (HuiShi and Mingyu Cao) and the full text was retrieved when the study was considered suitable for inclusion in this review. The evaluators also manually searched the reference lists of the included articles to identify possible studies that were not detected by the search strategy. If there was any disagreement, it would be resolved through discussion with a third researcher (YanZhao).

### Data extraction

For each included study, two reviewers (YanZhao and ChongTian) separately extracted following data: first author, year of publication, research aim and key findings, types of studies included in the systematic reviews, sample size and characteristics, interventions of the experimental group and the control group, outcome measures of and conclusion. Any discrepancies in data extraction were resolved through discussion between the two reviewers.

### Assessment of methodological quality

The methodological quality of the included studies was independently assessed by two reviewers (Songxin Tian and WeiHan) using the AMSTAR 2 tool [13]. AMSTAR 2 consists of 16 items to measure the methodological quality of SRs., of which item 2, 4, 7, 9, 11, 13 and 15 are considered critical (see in the Supplementary Materials 2), and the evaluation results are with three options of “Yes”, “Partially Yes” and “No”, and the quality of the literature is rated as “High”, " Moderate”, “Low” and " Critically low” according to the compliance of the entries. A third researcher (JieLiu) determined the results when there were disagreements.

### Assessment of quality of evidence

Two reviewers (Songxin Tian and WeiHan) independently used the Grading of Recommendations Assessment, Development, and Evaluation (GRADE) system [14] to evaluate the evidence quality of the meta-analyses included in this review. The GRADE rating classifies the quality of evidence into four levels: high, moderate, low, and very low, mainly in accordance with study design, risk of bias, inconsistency, indirectness, imprecision, and publication bias. The GRADE does not apply to qualitative evidence, so this study chose the Confidence in the Evidence from Reviews of Qualitative research (CERQual) to evaluate the degree of confidence of the results of qualitative evidence synthesis [15]. The CERQual is evaluated in accordance with four aspects: methodological limitations, the relevance of the research to the review question, consistency of the review findings, and adequacy of the data to support the review results. Then synthesizes the evaluation results of the above parts to give a reliable classification of the individual results of the system evaluation as “high, moderate, low, and very low”.

### Synthesis methods

Given the significant heterogeneity of interventions, outcome indicators and study designs in the included studies, this umbrella review chose to adopt a narrative synthesis approach. This approach allows for a clear and transparent summary of the findings across studies.

## Results

### Literature screening results

**Fig. 1:**
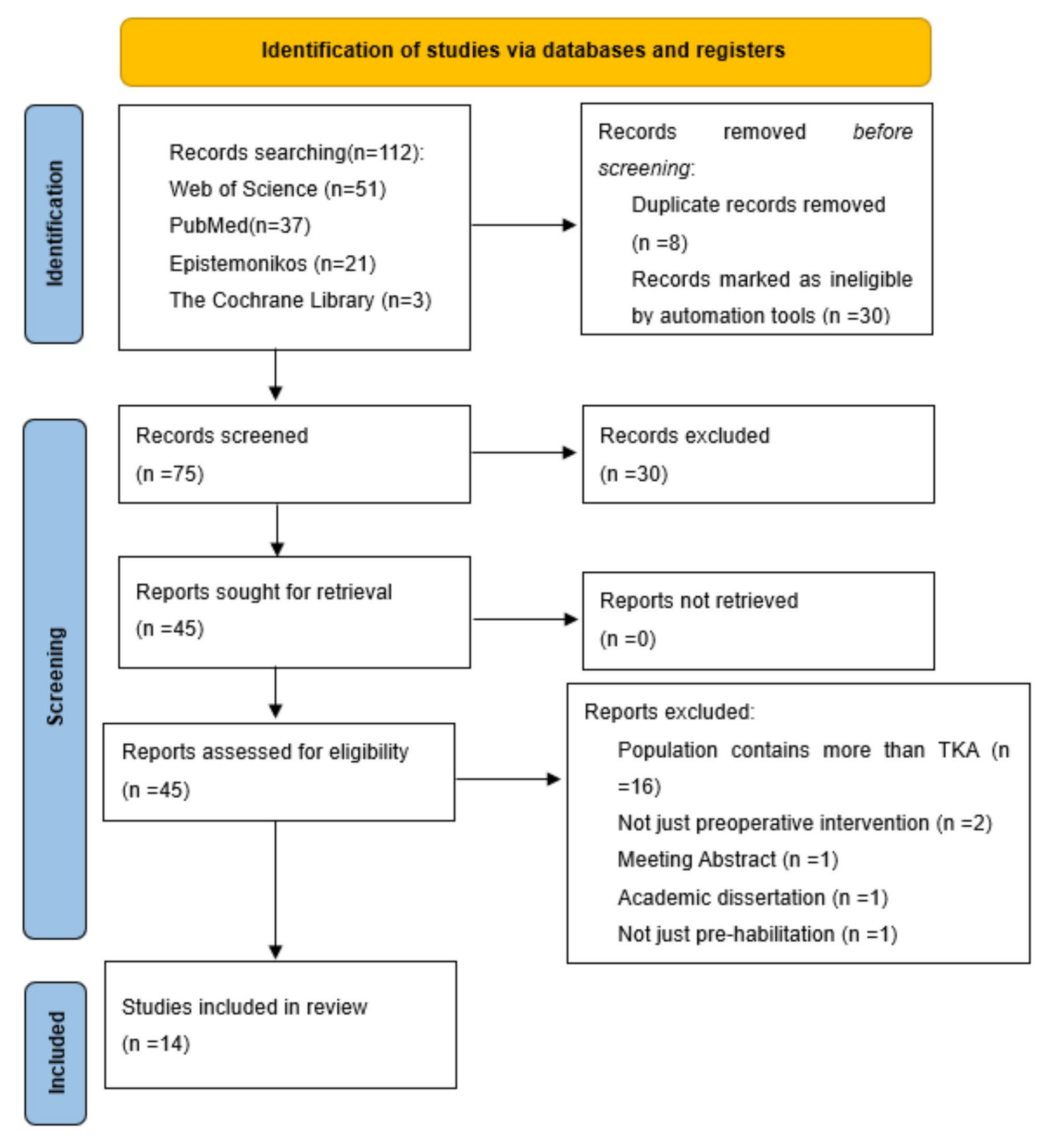
Flow chart of eligible studies

As shown in Fig. 1, a total of 112 articles were identified in this study, 75 of which were identified through the initial search. In addition, no other records were identified through other sources (*n* = 0). After further literature screening, 14 review articles were finally included [16–29], of which 4 were systematic evaluations [17, 18, 28, 29]. During the literature screening process, 21 studies failed to meet the inclusion criteria for the following reasons: Population contains more than TKA (*n* = 16), Not just preoperative intervention (*n* = 2), Academic dissertation (*n* = 1), Not just prehabilitation (*n* = 1), and conference abstracts (*n* = 1). The excluded studies and the detailed reasons for their exclusion are listed in Table S2 of the supplementary material. The excluded studies and their reasons for exclusion are detailed in Table S2 of the Supplementary Material.

### Research characteristics

The main characteristics of the 14 included reviews were shown in Table S3 in the Supplementary Material. The quantitative difference of original studies included in each SR varied greatly, ranging from 3 to 17. Most of the original studies were randomized controlled trials. Differences in research design also indirectly caused to distinct differences in the sample sizes, ranging from 82 to 1000. rehabilitation mainly supervised/unsupervised/home-based exercise and physiotherapy. The outcomes focus on length of hospital stay (LOS), postoperative pain, functional outcome and quality of life. Four studies [16, 20, 21, 27] clearly supported that preoperative rehabilitation could obviously shorten the length of hospital stay. Most studies do not have sufficient evidence to support that preoperative rehabilitation can improve patients’ pain, functional activity and quality of life.

### Assessment of methodological quality

**Fig. 2:**
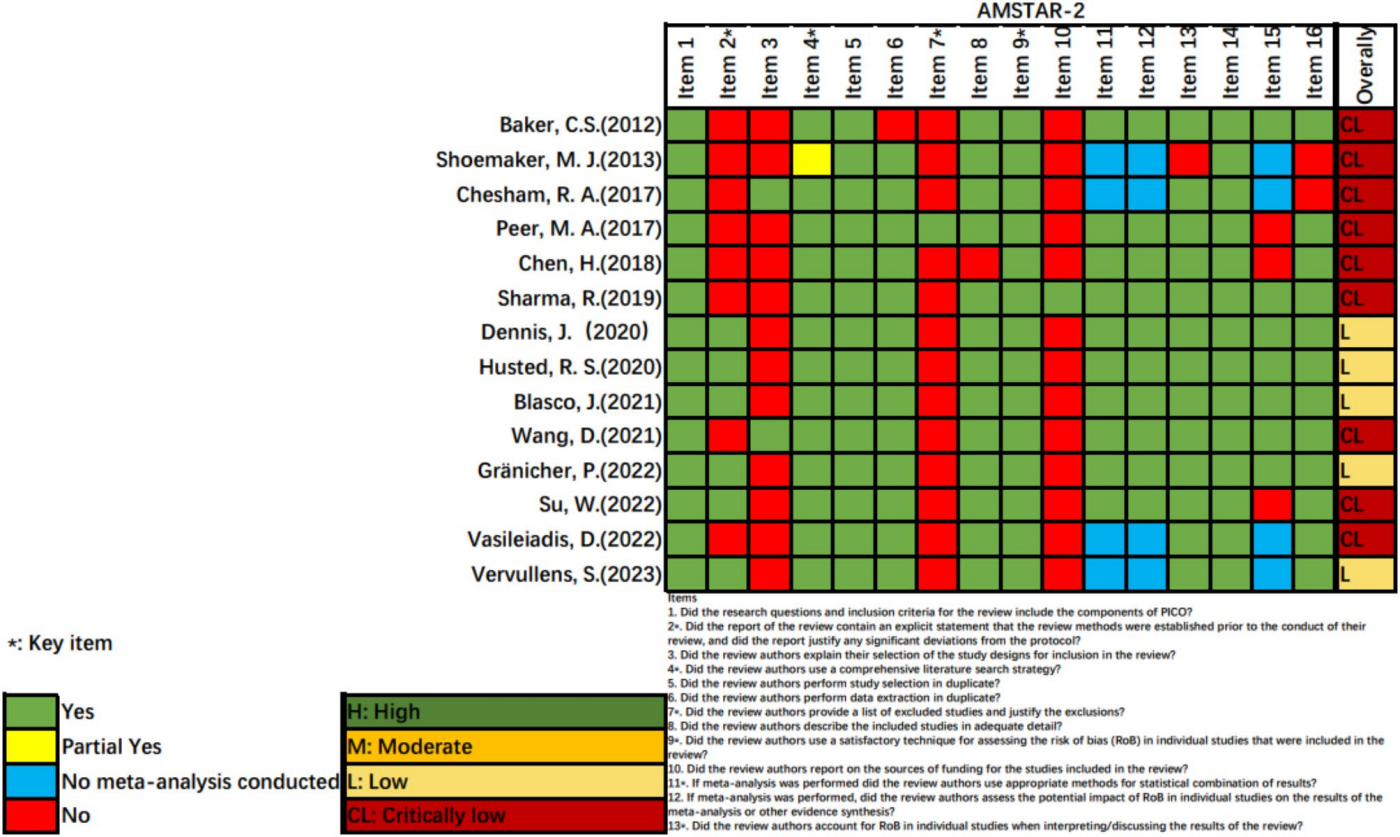
Methodological quality of the systematic reviews

According to the evaluation results of AMSTAR 2, the methodological quality of the 14 articles included was generally low. Among them, only 5 studies were low quality. However, the rest was critically low. Detailed results for each item in AMSTAR 2 are presented in Fig. 2. Key items 2 and 7 have more reporting defects. Others have more reported deficiencies for items 3 and 10.

### Assessment of quality of evidence

According to the evaluation results of GRADE, the 9 meta-analyses were low quality. All studies were downgraded for risk of bias and imprecision (Details can be seen in Table S4 in the Supplementary Material). The 4 qualitative systematic reviews were rated by CERQual as low quality (Table S5 in the Supplementary Material).

### Summary and analysis of evidence

By extracting and summarising the evidence for the rehabilitation of TKA patients, a total of 5 pieces of best evidence were extracted from3 aspects as shown in Table 1.

**Table 1:** Summary of preoperative rehabilitation evidence for patients undergoing TKA

Topic category	Description of evidence	Evidence grade	Recommended level
Shorten the length of hospital stay	Preoperative exercise [16, 20]; 3 sessions/week;3–8 weeks [16].	Low	A
	Preoperative exercise with/without patient education 1-3 day/week;4–8 weeks [21].	Low	A
	Preoperative physiotherapy (exercise and/or electrotherapy);2-5 day/week;3–12 weeks [27].	Low	A
Pain reduction	Preoperative physiotherapy (exercise and/or electrotherapy);2-7 day/week;3–12 weeks [28, 29].	Low	A
Increase knee range of motion (ROM)	Preoperative exercise [20, 25]; 2-5 day/week;3–12 weeks [25].	Low	A
	Preoperative exercise with/without patient education; 3 day/week;3-6weeks [26].	Low	A

## Discussion

A growing number of researches have gone to investigate the influence of TKA patients receiving preoperative rehabilitation on postoperative outcomes. In order to consolidate the evidence, more studies have generated systematic reviews and meta-analyses. However, the high methodological heterogeneity and unclear quality of evidence make clinical application difficult. The Umbrella Review overcomes this problem by assessing the methodological and evidence quality of published SRs in the field, providing a reliable overview of the impact of preoperative rehabilitation on postoperative outcomes. Umbrella reviews are more instructive when a patient’s rehabilitation plan needs to be developed clinically. Therefore, in this study, the best evidence for preoperative rehabilitation of TKA patients under rapid rehabilitation was extracted through a systematic search of the literature, screening and assessing the quality of the literature, with a total of 5 best evidence in 3 areas.

### Rehabilitation intervention

As research in the field of rehabilitation continues to evolve, the focus on preoperative optimization is gradually increasing. In order to ensure that patients receive the most effective treatment possible before surgery, rehabilitation management strategies necessitate multidisciplinary collaboration. Currently, the most commonly used protocol is ‘triple recovery’, a preoperative management strategy based on the concept of ERAS (Enhanced Recovery After Surgery), which includes nutritional (N), exercise (E) and psychological interventions (W) [4]. Numerous studies have shown that the optimization of preoperative nutritional status, physical function baseline and mental state can enhance the postoperative recovery ability of patients and shorten the hospitalization time of patients [30–34]. Preoperative nutritional, exercise, and psychological interventions are of particular importance for older patients, who often face increased stress due to low physiological reserves and high surgical risk. The findings of this study indicate that the prevailing systematic evaluation of knee arthroplasty patients places emphasis on exercise and physiotherapy, yielding marginal enhancements in postoperative pain and function. Moreover, there is a paucity of rehabilitation strategies targeting nutritional and psychological interventions. Consequently, the formation of a multidisciplinary team in clinical practice is recommended, with the objective of implementing a ‘triple rehabilitation’ programme that integrates preoperative nutritional, exercise and psychological interventions. The ultimate aim of this initiative is to promote the overall recovery of patients.

The absence of compelling evidence regarding the efficacy of the interventions currently incorporated into TKA rehabilitation research signifies a pivotal area for future investigation.

### Outcome measurement

This study clearly supports that preoperative rehabilitation significantly reduces the duration of hospitalization [16, 20, 21, 27]. The length of stay is defined as the number of days from the time of patient admission for TKA to the time of discharge. It is a metric that reflects the rate of functional recovery following surgery and whether the patient meets the discharge criteria [35]. The LOS of a patient is the primary factor contributing to the overall cost of the patient [36]. According to the 2000 data, the mean LOS in the hospital following TKA was 3.9 ± 1.9 days [36]. However, it is important to consider that factors like blood transfusion, complications, or advanced age could potentially extend the LOS after TKA [37, 38]. Therefore, preoperative individualized assessment of TKA patients, identification of potential risk factors and early development of a comprehensive preoperative rehabilitation programme are essential to facilitate early discharge.

In patients diagnosed with KOA, pain is a significant factor impacting the extent of knee flexion and extension. The primary objective of TKA is to alleviate pain. The present study found that KOA patients with an absence of regular exercise routines are often concerned that such activity will exacerbate joint damage. However, studies have shown that preoperative rehabilitation is effective in alleviating this concern [25, 26], helping patients find ways to cope with pain and maintain postoperative levels of function improves quality of life. In addition, this study identified a lack of evaluation of other preoperative interventions. For example, the lack of multimodal pain management may negatively impact long-term pain outcomes after TKA.

Knee mobility is an important physical function for patients with KOA, and limited knee mobility can greatly affect patients’ daily activities and quality of life. This study demonstrated that preoperative exercise intervention and/or patient education can effectively improve knee mobility, which is consistent with previous studies [20, 25, 26]. Therefore, rehabilitation interventions should be carried out as early as possible in clinical practice, depending on the patient’s specific situation, to ensure the maintenance of knee function.

### Methodological quality and bias

High-quality and low-risk studies are essential for clinical decision-making, and this review assessed the methodological quality of the included literature using the AMSTAR-2 tool. The results of the assessment showed that only 5 of the 14 reviews were rated as low quality, although the majority of the literature had significant methodological problems. The results of the AMSTAR-2 assessment suggest that the included literature generally suffers from a lack of study protocols and registration numbers, and a limited scope of searching, which can lead to biased study results. Registering study protocols helps to ensure the transparency of studies, reduces duplication and risk of bias, and facilitates the updating of study evidence [13]. Prior to conducting research on SRs, researchers are advised to check and register their study protocols on relevant registration platforms (e.g. PROSPERO, Cochrane, INPLASY, etc.), and also publicly via open journals or research institutions [12]. Some of the studies did not list in detail the reasons for exclusion from the literature, which may be subject to selective bias, and it is therefore recommended that exclusion criteria be provided through supplementary material. In addition, financial support and conflict of interest were not adequately reported in the included literature, and there was no mention of whether or not the original study received financial support, which affected the reader’s judgement of whether or not the results of the study might be biased.

### Quality of evidence

This study assessed the quality of evidence in the included literature using the GRADE and CERQual tools, and all literature was rated as low quality. The main reasons for the declining quality of evidence included insufficient methodological control, too small a number of original studies, and too small a sample size, which together led to imprecision and bias. To improve the quality of evidence, this study suggests that: first, original studies should be strictly controlled for bias during design and implementation; second, reviews should comprehensively obtain and synthesize relevant information from original studies; third, systematic evaluations should be updated in a timely manner with the release of new original studies; and finally, it is recommended that a diverse range of original studies should be conducted in different settings.

### Limitations and prospect

This study systematically assessed the most recent evidence on preoperative rehabilitation in improving postoperative outcomes in TKA, but there are some limitations. Firstly, this study only included studies that completed interventions preoperatively, excluding those that assessed interventions that started preoperatively and continued into the postoperative phase. Second, this study included only literature published in English, and therefore may have suffered from publication bias. Third, there were large differences in preoperative rehabilitation protocols across studies, which may have led to heterogeneity in postoperative functional outcomes.

In a recent literature search after September 2023, we identified three relevant studies. Of these, the first study demonstrated significant effects of prehabilitation in improving postoperative function, pain, muscle strength and quality of life after TKA, further supporting the findings of the present study [39]; the second study noted that a prehabilitation programme combined with lifestyle interventions (e.g., diet, stress management) demonstrated a positive trend in improving postoperative function and quality of life, but the evidence is still limited, suggesting that a more individualized intervention strategies [40]; a third study highlighted the effectiveness of prehabilitation as part of a fast-track programme in reducing length of stay and risk of postoperative complications, which provides new support for the practical application of this study [41]. Future studies need to further validate these findings and delve deeper into the mechanisms of action of prehabilitation, particularly in terms of optimization of intervention dose, duration and multimodal combinations.

## Conclusion

The articles included in this study have a relatively large heterogeneity in the choice of outcome indicators and interventions. Therefore, a small part of the evidence of low certainty suggests that rehabilitation in TKA patients may improve postoperative outcomes. Future research needs to address these issues through appropriate doses and fully described interventions before clinical recommendations are made on the model and implementation of TKA rehabilitation.

## Electronic supplementary material

Below is the link to the electronic supplementary material.

Supplementary Material 1

Supplementary Material 2

## Data Availability

This study has been registered with PROSPERO under registration number CRD420250654961, details of which can be accessed: https://www.crd.york.ac.uk/PROSPERO/.

## Abbreviations

KOA: Knee osteoarthritis
TKA: Total knee arthroplasty
ERAS: Enhanced recovery after surgery
PRISMA: Preferred Reporting Items for Systematic Reviews and Meta-Analyses
SRs: Systematic reviews and meta-analyses
AMSTAR-2: A Measurement Tool to Assess Systematic Reviews
GRADE: Grading of Recommendations Assessment, Development
CERQual: Confidence in the Evidence from Reviews of Qualitative research
LOS: Length of hospital stay
