# Supplementary material for: An umbrella review for preoperative rehabilitation in primary total knee arthroplasty: quality assessment and summary of evidence
Source: BMC Musculoskelet Disord. 2025 Jul 4;26:630. doi: 10.1186/s12891-025-08541-y (PMC12231914; doi:10.1186/s12891-025-08541-y)
Supplement: Supplementary file 2 — Supplementary Material 2 [file 12891_2025_8541_MOESM2_ESM.docx]

**Supplementary Material**

**Table S1**. Search strategy used for all searched databases

Literature search in PubMed, Web of Science, Epistemonikos and The Cochrane Library. Searches performed 2023-09-30.

| **Databases** | **Group** | **Search Syntax** | **Records** |
| --- | --- | --- | --- |
| **PubMed** | #1 | "Preoperative Exercise"[Mesh] OR "preoperative rehabilitation"[Title/Abstract] OR "perioperative care"[Title/Abstract] OR "prehabilitation recommendations"[Title/Abstract] OR "preoperative physical therapy"[Title/Abstract] OR "preoperative exercise"[Title/Abstract] | 6801 |
|  | #2 | "Arthroplasty, Replacement, Knee"[Mesh] OR "total knee replacement"[Title/Abstract] OR "total knee arthroplasty"[Title/Abstract] | 42183 |
|  | #3 | ("meta-analysis"[Title/Abstract]) OR ("meta-analyses"[Title/Abstract]) OR ("systematic review"[Title/Abstract]) OR ("systematic reviews"[Title/Abstract] ) OR (Meta-Analysis [Publication Type]) OR (systematic review[Publication Type]) | 475121 |
|  | #4 | #1 AND #2 AND #3 | 37 |
| **Web of Science** | #1 | TS=("preoperative rehabilitation" OR "perioperative care" OR "prehabilitation recommendations" OR "preoperative physical therap*" OR "preoperative exercise") | 13188 |
|  | #2 | TS=("total knee replacement" OR "total knee arthroplasty") | 26517 |
|  | #3 | TI=("meta-analysis" OR "meta-analyses" OR "systematic review" OR "systematic reviews") | 331352 |
|  | #4 | #1 AND #2 AND #3 | 51 |
| **Epistemonikos** | #1 | (title:("preoperative rehabilitation" OR "perioperative care" OR "prehabilitation recommendations" OR "preoperative physical therap*" OR "preoperative exercise") OR abstract:("preoperative rehabilitation" OR "perioperative care" OR "prehabilitation recommendations" OR "preoperative physical therap*" OR "preoperative exercise")) | 1349 |
|  | #2 | (title:("total knee replacement" OR "total knee arthroplasty") OR abstract:("total knee replacement" OR "total knee arthroplasty")) | 14636 |
|  | #3 | ("meta-analysis" OR "meta-analyses" OR "systematic review" OR "systematic reviews"):ti,ab,kw | 430950 |
|  | #4 | #1 AND #2 AND #3 | 21 |
| **The Cochrane Library** | #1 | ("preoperative rehabilitation" OR "perioperative care" OR "prehabilitation recommendations" OR "preoperative physical therap*" OR "preoperative exercise"):ti,ab,kw | 2361 |
|  | #2 | ("total knee replacement" OR "total knee arthroplasty"):ti,ab,kw | 8024 |
|  | #3 | ("meta-analysis" OR "meta-analyses" OR "systematic review" OR "systematic reviews"):ti,ab,kw | 26594 |
|  | #4 | #1 AND #2 AND #3 | 3 |

**Table S2.** Excluded reviews with the reasons for their exclusion

| **N** | **Author / Year** | **Title** | **Reasons for exclusion** |
| --- | --- | --- | --- |
| 1 | Haas, R., et al. (2016) | Early commencement of physical therapy in the acute phase following elective lower limb arthroplasty produces favorable outcomes: a systematic review and meta-analysis examining allied health service models | Population contains more than TKA |
| 2 | Liu, H., et al. (2021) | Efficacy and safety of lower-limb progressive resistance exercise for patients with total knee arthroplasty: a meta-analysis of randomized controlled trials | Not just preoperative intervention. |
| 3 | Health Quality, et al. (2005) | Physiotherapy rehabilitation after total knee or hip replacement: an evidence-based analysis | Population contains more than TKA |
| 4 | Coudeyre, E., et al. (2007) | Could preoperative rehabilitation modify postoperative outcomes after total hip and knee arthroplasty? Elaboration of French clinical practice guidelines | Population contains more than TKA |
| 5 | Bandholm, T., et al. (2012) | Physiotherapy exercise after fast-track total hip and knee arthroplasty: time for reconsideration? | Population contains more than TKA |
| 6 | Mak, J. C., et al. (2014) | Evidence-based review for patients undergoing elective hip and knee replacement | Population contains more than TKA |
| 7 | Skoffer, Birgit, et al. (2015) | Progressive resistance training before and after total hip and knee arthroplasty: a systematic review | Population contains more than TKA |
| 8 | Masaracchio, Michael, et al. (2017) | Timing of rehabilitation on length of stay and cost in patients with hip or knee joint arthroplasty: A systematic review with meta-analysis | Population contains more than TKA |
| 9 | Moyer, R., et al. (2017) | The Value of Preoperative Exercise and Education for Patients Undergoing Total Hip and Knee Arthroplasty: A Systematic Review and Meta-Analysis | Population contains more than TKA |
| 10 | Burgess, Louise C., et al. (2018) | What Is the Role of Nutritional Supplements in Support of Total Hip Replacement and Total Knee Replacement Surgeries? A Systematic Review | Population contains more than TKA |
| 11 | Yang, L., et al. (2019) | The effectiveness of air-free warming systems on perioperative hypothermia in total hip and knee arthroplasty A systematic review and meta-analysis | Population contains more than TKA |
| 12 | Wainwright, T. W., et al. (2020) | Consensus statement for perioperative care in total hip replacement and total knee replacement surgery: Enhanced Recovery After Surgery (ERAS(®)) Society recommendations | Population contains more than TKA |
| 13 | Wang, Peng, et al. (2020) | Analgesic effect of perioperative ketamine for total hip arthroplasties and total knee arthroplasties A PRISMA-compliant meta-analysis | Population contains more than TKA |
| 14 | Morrell, Aidan T., et al. (2021) | Enhanced Recovery After Primary Total Hip and Knee Arthroplasty A Systematic Review | Population contains more than TKA |
| 15 | Widmer, P., et al. (2022) | Effect of Prehabilitation in Form of Exercise and/or Education in Patients Undergoing Total Hip Arthroplasty on Postoperative Outcomes-A Systematic Review | Population contains more than TKA |
| 16 | Page, B. M., et al. (2023) | Impact of outpatient total hip or knee replacement on informal caregivers at home: a scoping review | Population contains more than TKA |
| 17 | Silkman, Carrie, et al. (2011) | Effects Of Preoperative Rehabilitation In Individuals Who Have Undergone Total Knee Arthroplasty: A Systematic Review | Meeting Abstract |
| 18 | Umehara, Takuya, et al. (2018) | Effective exercise intervention period for improving body function or activity in patients with knee osteoarthritis undergoing total knee arthroplasty: a systematic review and meta-analysis | Not just preoperative intervention. |
| 19 | Ferreira, SilvanaFonseca Z. (2021) | Effects of Implementing Exercise Programs on Patients Diagnosed with Gonarthrosis Prior to Total Knee Arthroplasty - A Systematic Review | Academic dissertation. |
| 20 | Hayashi, Kazuhiro, et al. (2023) | Preoperative Exercise Has a Modest Effect on Postoperative Pain, Function, Quality of Life, and Complications: A Systematic Review and Meta-Analysis | Population contains more than TKA |
| 21 | Beswick, A. D., et al. (2019) | Are perioperative interventions effective in preventing chronic pain after primary total knee replacement? A systematic review | Not just prehabilitation. |

**Table S3.** The main characteristics of the 14 included reviews.

| **No** | **Author / Year** | **Article type** | **Number of studies included** | **Inclusion in study design** | **sample size** | **Outcome variables** |
| --- | --- | --- | --- | --- | --- | --- |
| 1 | Baker, C.S.(2012) | Systemic review | 7 | Randomized control study design(n=2)  Non-randomized control study design(n=5) | 624 | Pain, stiffness, and physical function, Function Activities of Daily Living, Sport/Recreation, and Quality of Life, Joint mobility, postoperative length of stay (LOS) in the hospital |
| 2 | Peer, M.A. (2017) | Systematic review and meta-analysis of randomized controlled trials | 3 | Randomized control study design(n=3) | 82 | Length of hospital stay (LOS), postoperative pain, functional outcome and quality of life |
| 3 | Chen, H.(2018) | Meta-analysis of randomized controlled trials | 16 | Randomized control study design(n=16) | 1224 | Length of hospital  stay, quadriceps strength and functional ability in short  term (1.5 to 3 months) after TKA |
| 4 | Sharma, R. (2019) | Systemic review | 5 | Randomized control study design(n=3) | 494 | Patients’ function, acute care length of stay (LOS), pain, and stiffness |
| 5 | Dennis, J.（2020） | Systematic review and meta-analysis of randomized controlled trials | 8 | Randomized control study design(n=8) | 960 | Pain at 6months or  longer, adverse events |
| 6 | Husted, R. S. (2020) | Systematic review and meta-regression analysis of randomized controlled trials | 12 | Randomized control study design(n=12) | 616 | Knee-extensor strength, knee pain, patient reported physical function (e.g., activities of daily living), knee-related performance-based function (e.g., ability to climb stairs) and adverse events |
| 7 | Blasco, J.M. (2021) | Systematic review and meta-analysis | 7 | Randomized control study design(n=7) | 332 | Balance,  pain, and quality of life |
| 8 | Wang, D. (2021) | Systematic review and meta-analysis | 12 | Randomized control study design(n=12) | 889 | (I) Range of Motion, ROM; (II) The Western Ontario and McMaster Universities Osteoarthritis Index (WOMAC) (6); (III) Visual Analogue Scale (VAS); (IV) Timed Up and Go (TUG) test; (V) 6-Minute Walk Test (6 MWT); (VI) Stair Climbing Test (SCT); (VII) quadriceps strength; (VIII) The Short Form of Health Survey Questionnaire (SF-36); (IX) the Knee Injury and Osteoarthritis Outcome Score (KOOS), and (X) the Berg Balance Scale (BBS) |
| 9 | Su, W. (2022) | Meta-analysis of randomized controlled trials | 19 | Randomized control study design(n=19) | 1008 | The major outcomes included pain, knee fexion and extension, as well as knee range of motion (ROM). Secondary outcomes included timed-up-and-go (TUG), 6-min walk, and patient-reported functional outcome (the Knee  Injury and Osteoarthritis Outcome Score (KOOS) or Western Ontario and McMaster Universities Osteoarthritis Index (WOMAC)). Third outcomes included the length of hospital stay. |
| 10 | Gränicher, P. (2022) | Systematic review and meta-analysis | 16 | Randomized control study design(n=16) | 968 | The primary outcome of interest was knee functioning, as defined by the International Classification of Functioning, Disability, and Health (ICF) model47,76,80 and measured via patient-reported outcome measures (PROMs) (eg, Knee Osteoarthritis Outcome Score [KOOS] and Function Score [FS]) and physical performance tests (eg, strength and range of motion [ROM]). |
| 11 | Shoemaker, M.J.(2013) | Systematic review and meta-analysis | 178 | Randomized control study design(n=13)  retrospective cohort study(n=1)  prospective case series(n=2)  case report(n=1) | 843 | Impairment-based outcome measures, Activity-based outcome measures, Participation-based outcome measures, Health care utilization outcomes |
| 12 | Chesham, R.A.(2017) | Systematic review | 10 | Randomized control study design(n=10) | Not reported | Knee strength, ambulation, and pain |
| 13 | Vasileiadis, D.(2022) | Systematic review | 24 | Randomized control study design(n=24) | 1499 | Knee extension, knee flexion, pain Visual Analogue Scale (VAS), overall Western Ontario and McMaster Universities OA Index, 6 min walking test, and Timed Up and Go test |
| 14 | Vervullens, S.(2023) | Systematic review | 17 | Prospective non-randomized controlled study(n=2)  Randomized control study design(n=15) | 1634 | Pain, satisfaction, function and quality of life |

**Table S4.** Results of the Grading of Recommendations Assessment, Development and Evaluation (GRADE) assessment

| **Reference** | **GRADE items** | | | | | **Quality of the evidence*** |
| --- | --- | --- | --- | --- | --- | --- |
|  | **Risk of bias** | **Inconsistency** | **Indirectness** | **Imprecision** | **Publication bias** |  |
| Baker, C.S.  (2012) | Serious | Not serious | Not serious | Serious | Undetected | Low  ⊕⊕ΟΟ |
| Peer, M.A. (2017) | Serious | Not serious | Not serious | Serious | Undetected | Low  ⊕⊕ΟΟ |
| Chen, H.  (2018) | Serious | Not serious | Not serious | Serious | Undetected | Low  ⊕⊕ΟΟ |
| Sharma, R. (2019) | Serious | Not serious | Not serious | Serious | Undetected | Low  ⊕⊕ΟΟ |
| Dennis, J.（2020） | Serious | Not serious | Not serious | Serious | Undetected | Low  ⊕⊕ΟΟ |
| Husted, R. S. (2020) | Serious | Not serious | Not serious | Serious | Undetected | Low  ⊕⊕ΟΟ |
| Blasco, J.M. (2021) | Serious | Not serious | Not serious | Serious | Undetected | Low  ⊕⊕ΟΟ |
| Wang, D. (2021) | Serious | Not serious | Not serious | Serious | Undetected | Low  ⊕⊕ΟΟ |
| Su, W.  (2022) | Serious | Not serious | Not serious | Serious | Undetected | Low  ⊕⊕ΟΟ |
| Gränicher, P. (2022) | Serious | Not serious | Not serious | Serious | Undetected | Low |
| * classification based on the GRADE Handbook as:  ⊕⊕⊕⊕ = high quality  ⊕⊕⊕Ο = moderate quality  ⊕⊕ΟΟ = low quality  ⊕ΟΟΟ = very low quality | | | | | | |

**Table S5.** Results of the Confidence in Evidence from Reviews of Qualitative research (CERQual) assessment

| **Reference** | **CERQual items** | | | | **Overall CERQual Assessment of Confidence** |
| --- | --- | --- | --- | --- | --- |
|  | **Assessment of Methodological Limitations** | **Assessment of Relevance** | **Assessment of Coherence** | **Assessment of Adequacy** |  |
| Shoemaker,M.J.(2013) | Moderate methodological limitations | No or very minor concerns about relevance | Serious concerns about coherence | Moderate concerns about adequacy | Low |
| Chesham, R.A.(2017) | Minor methodological limitations | No or very minor concerns about relevance | Serious concerns about coherence | Moderate concerns about adequacy | Low |
| Vasileiadis, D.(2022) | Minor methodological limitations | No or very minor concerns about relevance | Serious concerns about coherence | Moderate concerns about adequacy | Low |
| Vervullens, S.(2023) | Moderate methodological limitations | No or very minor concerns about relevance | Serious concerns about coherence | Moderate concerns about adequacy | Low |
